# Regulation of Enzyme Activity through Interactions with Nanoparticles

**DOI:** 10.3390/ijms10104198

**Published:** 2009-11-20

**Authors:** Zhaochun Wu, Bin Zhang, Bing Yan

**Affiliations:** 1 School of Pharmaceutical Sciences, Shandong University, Jinan 250012, China; 2 School of Chemistry and Chemical Engineering, Shandong University, Jinan 250100, China; 3 St. Jude Children’s Research Hospital, Memphis, TN 38105, USA

**Keywords:** enzyme activity, regulation, nanoparticle

## Abstract

The structure and function of an enzyme can be altered by nanoparticles (NPs). The interaction between enzyme and NPs is governed by the key properties of NPs, such as structure, size, surface chemistry, charge and surface shape. Recent representative studies on the NP-enzyme interactions and the regulation of enzyme activity by NPs with different size, composition and surface modification are reviewed.

## 1. Introduction

The regulation of the protein activity plays an important role in modulating cellular processes such as signal transduction, DNA replication, and metabolism [1,2]. Protein dysfunction is related to human diseases and disorders [3–7], and our ability to regulate enzyme functions and protein-protein interactions provides a promising strategy for therapy. Nanoparticles (NPs) have some advantages over small organic molecules. First, NPs have large specific surface areas for adequate protein binding and biological interactions [1,8]. Second, NP can enter cells easily [10], in contrast to some small molecules and biological molecules. Third, there has been considerable progress in the synthesis of NPs with well controlled dimensions, geometry, and surface properties [9], to complement the structural complexity of proteins [11,12]. Recent developments in nano materials offer a new pathway for controlling protein behavior through surface interactions.

In the past few years, NPs with different dimensions, composition and surface modification were investigated to understand their interactions with proteins. The hydrophobic interaction [13–15], π-π stacking [16–18] and electrostatic interaction [19–21] have been attributed to be major mechanisms of NP-protein interactions. These mechanisms often coexist.

This review, by summarizing recent research on nanoparticle/protein interactions, intends to emphasize the importance of such interactions in biological systems that may cause nanotoxicity issues, and the potential of such molecular recognition events in biomedical applications such as the diagnosis and treatment of human diseases.

## 2. Effects of Mixed Monolayer Protected Nano Clusters (MMPCs)

**Figure 1.**
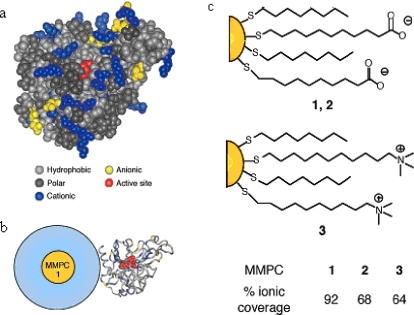
(a) Space-filling model of ChT. Surface binding of the proteins by anionic MMPCs focuses on the ring of cationic residues situated around the active site, functionally significant residues are noted. (b) Relative sizes of ChT and MMPC **1**. (c) Anionic **MMPCs 1** and **2** and cationic control **3** (reproduced with permission from [25] ^©^ 2004, National Academy of Sciences, U.S.A).

Mixed monolayer protected gold clusters (MMPCs) provide an effective scaffold for biomolecular binding. MMPCs were synthesized using the Brust reduction and Murray place-displacement reaction [24] to fabricate additional functionalized thiols ended with carboxylate groups and amino groups, respectively [MMPCs **1**, **2**, and **3** in Figure 1(c) featuring a 2-nm gold core, with an overall diameter of 6 nm] [25]. These particles exhibited different effects on chymotrypsin (ChT) activity. The cationic MMPC **3** had no inhibition, while the anionic MMPC **1** and **2** were effective inhibitors of ChT because of the electrostatic complementarity between the carboxylate end groups and the hole of cationic residues located around the periphery of the active site, as shown in Figure 1(b). Complete inhibition was observed at a 1:5 nanoparticle to ChT ratio.

The activity assay indicated that the inhibition of ChT by MMPCs was controlled by a two-stage mechanism featuring a fast reversible inhibition, followed by a slower irreversible process. Circular dichroism measurements of the complex demonstrated an almost complete denaturation of the enzyme over time. Dynamic light scattering studies confirmed that inhibition proceeded without substantial MMPC aggregation. The electrostatic nature of the engineered interactions provides a level of selectivity: little or no inhibition of functions of elastase, β-galactosidase, or cellular retinoic acid binding protein by MMPC was observed.

**Figure 2.**
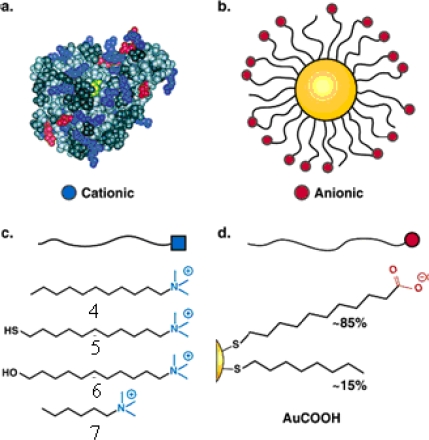
(a) Space-filling model of ChT. Active site (yellow) is surrounded by a ring of cationic residues (blue). (b) Amphiphilic MMPCs with anionic functional groups (red) in their terminals can interact with ChT via electrostatic complementarity. (c) Four derivatives of trimethylamine-functionalized surfactant used for surface modification of MMPC anionic monolayer. (d) Monolayer composition of AuCOOH (reproduced with permission from [26] ^©^ 2003, American Chemical Society).

**Figure 3.**
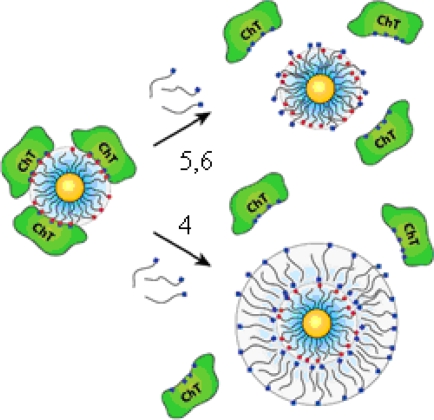
Proposed mechanism of ChT rescue. The surfactants with thiol **5** and alcohol **6** at their terminals have the ability to elict ChT release by interaction and/or partial displacement of the anionic monolayer. C_11_ alkane **4** forms a bilayer-like structure, causing release of the ChT (red = anionic; blue = cationic) (reproduced with permission from [26] ^©^ 2003, American Chemical Society).

The “irreversible” inhibition of ChT can be reversed through modification of the anionic MMPC surface by the addition of cationic surfactants [26]. Four derivatives of trimethylamine-functionalized surfactants were used to modifiy the gold nanoparticles (GNPs) with carboxylate endgroups (**4**, **5**, **6** and **7** in Figure 2c, featuring a 2-nm gold core, with an overall diameter of 6 nm). Up to 50% of original ChT activity was rescued upon long-chain surfactant addition. Dynamic light scattering studies demonstrated that ChT released from the nanoparticle surface and the conformation characterization of the rescued ChT by fluorescence and fluorescence anisotropy indicated that ChT regained a high degree of native structure upon surfactant addition. The proposed mechanism of the ChT release: the MMPC **5** and MMPC **6** can elicit ChT release by interaction and/or partial displacement of the anionic monolayer. The MMPC **4**, a bilayer-type structure, can release ChT when it envelops the MMPC surface, as shown schematically in Figure 3. The noncovalent nature of the irreversible inhibition of ChT shows that the attenuation of the interactions the MMPC and protein could provide a means of rescuing enzyme activity.

## 3. Influence of the Size of NP on the Activity of Adsorbed Enzymes

**Figure 4.**
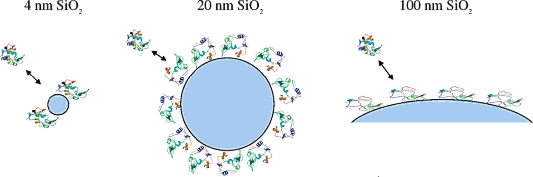
Schematic of lysozyme adsorption on silica particles with different sizes. Stronger protein particle interactions exist in the case of larger nanoparticles, resulting in more protein unfolding and less enzymatic activity (reproduced with permission from [27] ^©^ 2004, American Chemical Society).

The size of NPs is one of the key parameters that influence the interaction between protein and NPs. Silica NPs with different diameters (4, 20 and 100 nm) were used to investigate the size influence on the structure and enzymatic activity of adsorbed lysozyme, whose dimensions are comparable in size to the 4 nm NPs [27]. Both adsorption patterns and protein structure and function are strongly dependent on the size of the NPs. The formation of molecular complexes is observed for adsorption onto 4-nm silica. The CD results proved that the loss in α-helix content is strongly dependent on the size of the NPs. The great loss of α helicity was observed for the lysozyme adsorbed onto larger NPs. The activity of lysozyme adsorbed onto silica NPs is lower than that of the free protein, and the fraction of activity lost correlates well with the decrease in α-helix content. These results indicate that the size of the NP, perhaps because of the contributions of surface curvature, influences adsorbed protein structure and function (Figure 4).

The same phenomenon was also observed when ribnuclease A [28] and human carbonic anhydrase [29] were absorbed onto silica NPs surfaces with different sizes. Urea denaturation analyses showed that the thermodynamic stability of ribnuclease A was decreased upon adsorption on to the NPs, with greater decrease on larger NPs. Therefore, the larger NPs tend to cause unfolding of adsorbed proteins [28].

## 4. Conformational Changes as a Result of NP/Protein Binding

Applications from diagnostics to drug delivery require that enzymes retain their native structures and activities. Conjugates of acid-treated SWNTs with three functionally unrelated enzymes horseradish peroxidase, subtilisin Carlsberg, and chicken egg white lysozymes were found to be soluble in aqueous solutions [30]. Enzyme kinetics revealed that these enzymes retained a high fraction of their native structures and activities. The SWNT-enzyme conjugates were also more stable in guanidine hydrochloride (GdnHCl) and at elevated temperatures relative to their solution counterparts. These studies demonstrate the enhanced stability of SWNT-enzyme preparations relative to free enzymes in solution. Such water-soluble enzyme-nanotube conjugates may be useful in a wide range of applications.

**Figure 5.**
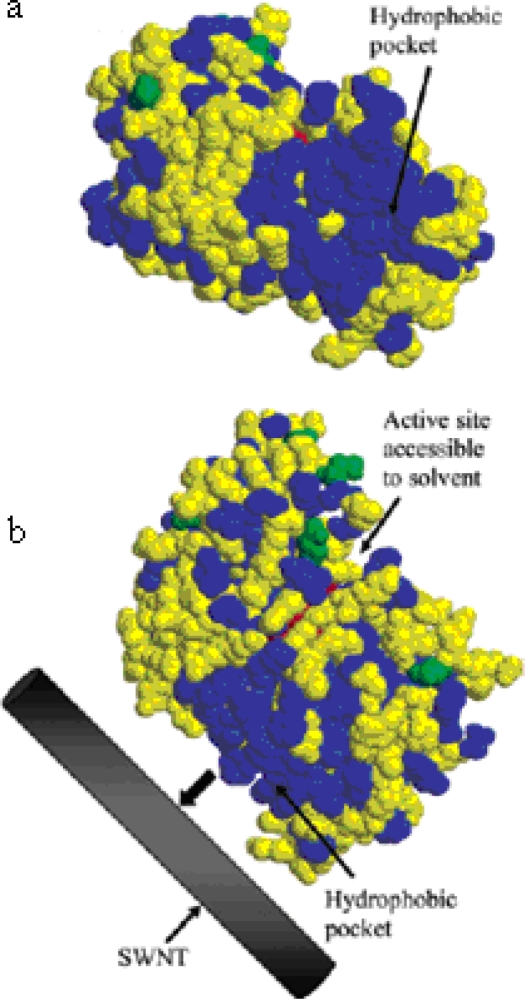
(a) PDB structure of SBP showing the hydrophobic pocket. Hydrophobic and hydrophilic residues are shown in blue and yellow, respectively, while the asparagines residues that are glycosylated are shown in green and the heme is shown in red. (b) A schematic hypothesizing the adsorption of SBP onto SWNTs via the hydrophobic pocket on SBP (reproduced with permission [31] ^©^ 2004, American Chemical Society).

Two enzymes, α-chymotrypsin and soybean peroxidase, were adsorbed onto the single-walled carbon nanotubes. α-chymotrypsin retained only 1% of its native activity while soybean peroxidase retained 30% of its native activity. The FT-IR spectroscopy revealed that substantial secondary structural perturbation is occurred after ChT binding to the SWNTs. This phenomenon was caused by the hydrophobic interaction between the SWNTs and the proteins. Soybean peroxidase had a hydrophobic pocket that bound to SWNTs leaving the active site exposed to the solvent to bind substrate. Denaturation of ChT was responsible for low activity (Figure 5) [31]. If the nanomaterial surface is modified with hydrophilic polymers such as oligomeric PEG moieties, the interactions of NPs and proteins may be alleviated [32].

## 5. Regulation of Enzyme Function by Surface-Modified NPs

Compared with traditional materials, NPs have a larger surface area that can be modified with organic molecules through covalent or non-covalent modifications to form functionalized NPs. The suitably functionalized NPs possess the abilities that prinstine NPs do not have, such as preventing non-specific bindings and recognizing specific biomacromolecules. In this way, enzyme activities can be specifically regulated when bound to surface modified NPs.

Three silicon nanowires (SiNW) [33], unmodified silicon nanowires (SiNW-SiO_2_), SiNWs functionalized with carboxylic groups (SiNW-COOH) and highly reactive hydrogen modified SiNW (SiNW-H) were synthesized to investigate their affect on restriction endonucleases and Taq DNA polymerase. The PCR results showed that the inhibition of enzyme activity was determined by the functional groups on SiNWs in the order SiNW-H > SiNW-COOH > SiNW-SiO_2_.

**Figure 6.**
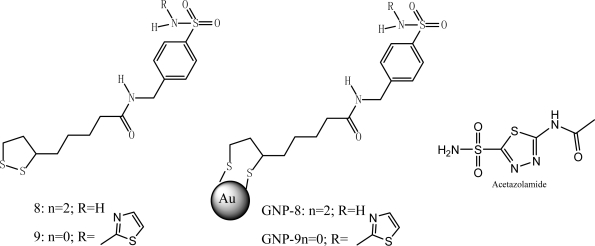
Structure of the acetazolamide AZA, the new sulfonamides **8**, **9**, **GNP-8** and **GNP-9.**

Specific NP/protein binding provides a better opportunity for regulating enzyme functions. Carbonic anhydrase inhibitor **8** linked to gold NPs (GNP**-8,** with an average particle size of 3.3 nm, which corresponds to 720–724 Au atoms) showed excellent CA IX inhibition of the tumer-associated isoform and selectivity over hCA I and II (Table 1, Figure 6) [34] due to the fact that their binding pocket contains different residues. Table 1 listed the inhibition of CA I, II and IX^a^ by acetazolamide (AZA), sulfonamides **8**, **9**, **GNP-8** and **GNP-9**. It has been also shown that molecules such as antibodies, which can recognize protein specifically, can be linked to NPs to recognize proteins through specific antibody-antigen interactions [35,36].

**Figure 7.**
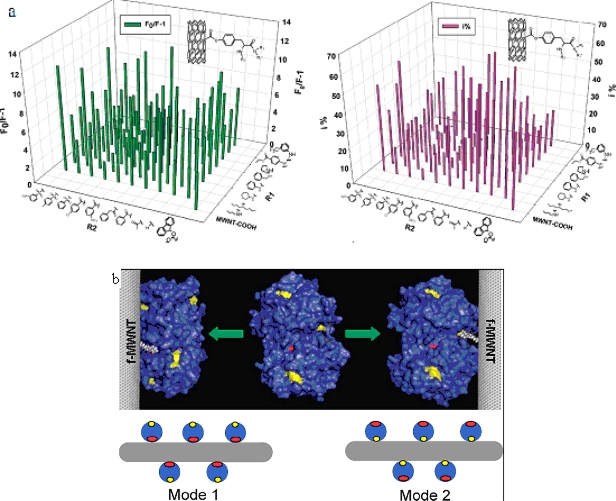
(a) The quenching of ChT’s fluorescence (left) and inhibition of its enzymatic activity (right) by f-MWNTs. (b) Models showing different ways f-MWNTs bind to ChT (reproduced with permission from [38] ^©^ 2004, American Chemical Society).

We have synthesized a novel surface-modified multi-wall carbon nanotube combinatorial library containing 80 members by using *in silico* design and combinatorial synthesis [37]. By screening the library we discovered MWNTs with reduced protein binding, reduced cytotoxicity, and immune response. The ChT enzymatic activity and fluorescent quenching were measured by incubating ChT with functionalized multiwalled carbon nanotube (f-MWNTs, were synthesized by chemical vapor deposition. The purity was 95% and the catalyst residue was less than 0.2%. They are 40 nm in diameter and the length is 500–2,000 nm) (Figure 7A). By screening the library [38], we discovered four f-MWNTs that can bind to the catalytic site of ChT site-specifically and inhibit its enzymatic activity competitively (Figure 7B). Our results demonstrated that specific recognition of ChT and regulation of its functions by surface-modified MWNT.

## 6. Fine Tuning of NP/Protein Interactions

**Figure 8.**
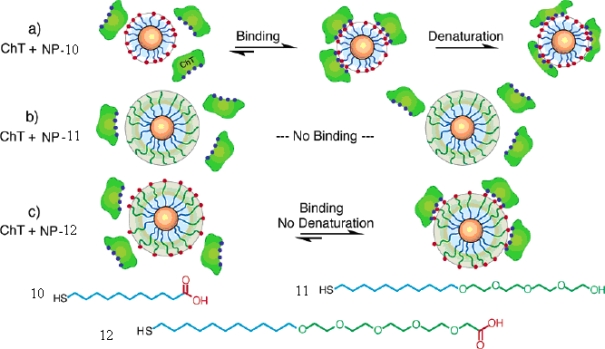
Ligands used for CdSe nanoparticles, and schematic depiction of protein-nanoparticle interactions (reproduced with permission from [39] ^©^ 2004, American Chemical Society).

CdSe particles modified with thioalkylated oligo(ethylene glycol) and chain-end carboxylate were synthesized [39]. The study of the interactions between CdSe NPs and chymotrypsin revealed three levels of inhibition: (1) protein inhibition and denaturation with **NP-10**, (2) no protein binding with **NP-11**, and (3) inhibition and retention of the protein structure with **NP-12**. The **NP-10** with alkanethiol-carboxylate functionalized can bind, inhibit, and denaturate of ChT because of the hydrophobic interactions. The **NP-11**, lacking of the carboxylate recognition element, was inert in protein binding. The **NP-12**, modified with tetraethylene glycol spacer between the alkyl chain and recognition element, can inhibit ChT reversibly because of the electrostatic interaction between the particle and protein, but prevents hydrophobic interactions caused by the interior alkyl chain (Figure 8).

In order to explore how the linkages between recognition elements and NP core affect the NP/enzyme interacion. A series of l-amino acid functionalized GNPs with oligo (ethylene glycol) tethers of varying length were studied. It has been shown that amino acid side chains can maintain the ChT structure while the alkyl chains denature the protein as a result of nonspecific hydrophobic interactions [40].

Structure diversity can also be generated by introducing amino acids in the surface modification. The hydrophobic interaction and the complementary electrostatic interactions between Au nanoparticles with l-amino acids as endgroups and ChT also plays an important role in regulating ChT activity [41].

Protein-protein recognition is a key aspect of the complex cellular functions, such as apoptosis and angiogenesis. Control over interprotein recognition holds the potential in therapeutic applications. Thiolates with biocompatible PEG linker and trimethyl-amine end group were used to functionalize Au nanoparticles. And it can inhibit interactions between cytochrome c and cytochrome c perxidase in the low nM concentration range [42].

## 7. Conclusions

Enzyme dysfunction is related to human diseases. It is desirable to be able to regulate enzyme conformation and function. Nano sensors incorporating enzymes also require that protein conformation is not altered, further emphasizing the importance of protein regulation. NPs can be selected to specifically bind enzymes and control their functions after surface modifications. Such NP/protein interactions can be fine-tuned to maintain protein structure or alter it on purpose.

## Figures and Tables

**Table 1.** CA inhibition data against isoforms CA I, II and IX a

	**K_i_^b^ (nM)**		
**Compound**	**hCA I**	**hCA II**	**hCA IX**
**AZA**	250 ± 12	12 ± 1	25 ± 1
**8**	214 ± 9	230 ± 10	41 ± 2
**GNP-8**	581 ± 18 (128)	451 ± 21 (116)	32 ± 2 (2.4)
**9**	>50,000	>50,000	>50,000
**GNP-9**	28,550	30,400	31,050

**Au**	32,000	31,600	29,560

a Reproduced with permission from [34] ^©^ 2003, American Chemical Society.

b Inhibition constants (K_i_) are obtained by nonlinear least-squares methods after being incubated with compound 15min.Data in parentheses show the inhibition constants when enzyme and inhibitor were incubated for 2 h.
